# Flora of Ferruginous Outcrops Under Climate Change: A Study in the *Cangas* of Carajás (Eastern Amazon)

**DOI:** 10.3389/fpls.2021.699034

**Published:** 2021-09-07

**Authors:** Tereza Cristina Giannini, André Luis Acosta, Wilian França Costa, Leonardo Miranda, Carlos Eduardo Pinto, Maurício Takashi Coutinho Watanabe, Daniela Cristina Zappi, Ana Maria Giulietti, Vera Lucia Imperatriz-Fonseca

**Affiliations:** ^1^Instituto Tecnológico Vale, Belém, Brazil; ^2^Programa de Pós Graduação em Zoologia, Universidade Federal do Pará, Belém, Brazil; ^3^Faculdade de Computação e Informática, Universidade Presbiteriana Mackenzie, São Paulo, Brazil; ^4^Museu Paraense Emílio Goeldi, Belém, Brazil; ^5^Departamento de Química Fundamental, Centro de Ciências Exatas e da Natureza, Universidade Federal de Pernambuco, Recife, Brazil; ^6^Programa de Pós-Graduação em Botânica, Instituto de Ciências Biológicas, Universidade de Brasília, Brasília, Brazil; ^7^Programa de Pós-Graduação em Botânica, Universidade Estadual de Feira de Santana, Feira de Santana, Brazil; ^8^Departamento de Ecologia, Instituto de Biociências, Universidade de São Paulo, São Paulo, Brazil

**Keywords:** Amazon biome, biodiversity, canga, conservation, mutualism, plant-pollinator interaction

## Abstract

Climate change has impacted biodiversity, affecting species and altering their geographical distribution. Besides understanding the impact in the species, it has been advocated that answering if different traits will be differently impacted could allow refined predictions of how climate change will jeopardize biodiversity. Our aim was to evaluate if climate change will potentially impact plant species differently, considering their traits. We evaluated 608 plant species that occur in the naturally open areas of ferruginous outcrops (namely, *cangas*) in the National Forest of Carajás (Eastern Amazon). Firstly, we estimated the effects of climate change on each species using species distribution modeling, and analyzed this impact in the set containing all species. Secondly, we classified plant species considering the following traits: (i) pollination syndromes (melittophily, phalaenophily, psychophily, cantharophily, entomophily, ornithophily, chiropterophily, anemophily); (ii) habit (tree, shrub, herb, liana, parasite); and (iii) the main habitat of occurrence (open areas and forests). Thirdly, we investigated if the effects of climate change could be significantly more intense considering all the different traits quoted. Our results showed that most plant species will potentially face reduction of suitable habitats under future climate and the scenarios showed that 42% of them may not find suitable areas in the cangas of Carajás. We found no significant difference within each analyzed trait, considering the potential impact of climate change. The most climatically suitable areas (i.e., areas with high probability of species occurrence in the future) are those in the southwest of the study area. These areas can be considered as priority areas for species protection against climate change.

## Introduction

Climate change due to anthropogenic activities has had negative effects on biodiversity, especially due to its unprecedented speed (Pacifici et al., 2015; Venter et al., 2016). The flora may be affected by several factors, including physiological and adaptive incompatibility with the new climatic conditions (Ahuja et al., 2010), reduced capacity or inability to disperse to new habitats (Corlett and Westcott, 2013), or negative morphological and phenological changes (Cleland et al., 2007). Moreover, climate change can alter the original geographic distribution of species. For example, changes in the average altitude of occurrence of plants have been observed (Kelly and Goulden, 2008; Lenoir et al., 2008; Steinbauer et al., 2018), as have changes toward new latitudes (Chen et al., 2011). Thus, anticipating the potential impact of climate change on plant species, especially considering different traits, is urgently need to effective conservation strategies.

Species traits are any characteristic of an individual that can be assigned to a species, which could be phenological, morphological, physiological, reproductive, or behavioral (Kissling et al., 2018). They were also defined as a measurable property of organisms; more specifically, a functional trait is one that strongly influences organismal performance, and it can provide important information about the species performance, especially considering global changes (McGill et al., 2006).

One example of a set of traits related to reproductive success is pollination syndrome. It relies on a specific set of traits (Dellinger, 2020), such as the shape, color, odor, nectar amount, and pollen location (Faegri and Van Der Pijl, 1979; Fenster et al., 2004). The concept of pollination syndrome is based on the assumption that flowers are adapted to their most efficient pollinator group (Dellinger, 2020), with floral morphology being an important factor that restricts the number of possible interactions with pollinators (Stang et al., 2007). Despite the common criticism to pollination syndrome, based mainly on the fact that many plants can be pollinated by different pollinators (Ollerton et al., 2009), high accuracy on pollinator’s prediction was found even when based on only a few traits, showing that it can accurately circumscribe plant-pollinator interactions (Dellinger, 2020). However, as far as we know, there is no study that addressed the impact of climate change considering different pollination syndromes.

Habit is an important morphological trait (Panchen et al., 2014). There is also no study comparing the impact of climate change on this trait in a large number of species. However, it was showed that global warming altered vegetation functional structure, considering fast-growing species and two different growth-habits (erect and prostate) (Debouk et al., 2015). The influence of harsh condition in the plant size was also demonstrated to a Pyrenean saxifrage (*Saxifraga longifolia* Lapeyr.), where larger plants showed higher resistance to stress (Cotado and Munné-Bosch, 2020). When comparing different growth habits of *Nothofagus pumilio* (Poepp. & Endl.) Krasser under an altitudinal gradient, a study showed that at high altitude, juvenile shrubby growth habits are favored, probably due to cold stress effects (Soliani and Aparicio, 2020).

Additionally, a third characteristic that was still scarcely addressed, when comparing the climate change impact on large set of plant species, is their preferential habitat. For example, the richness of trees occurring in the tropical Amazon forest are expected to reduce by 31–37% (year 2050) under scenarios of climate change, and tree species can potentially lose 65% of their environmentally suitable area (Gomes et al., 2019). Considering the woody plant assemblage occurring in the Atlantic Forest, it was projected an overall reduction of its beta diversity under future climatic scenario (Zwiener et al., 2018). As for the Brazilian cerrado vegetation, an analysis of 1,553 plant species showed that species will lose an average of 34–40% (under the optimistic scenario) and 43–60% (under the pessimistic scenario) of their suitable occurrence area (Velazco et al., 2019).

National Forest of Carajás is one of the few remaining natural habitats located in the eastern Amazon, an area highly affected by deforestation (Souza-Filho et al., 2016). Rock outcrops presenting a high iron concentration and shallow soil occur within the Amazon forest and are locally known as canga (Mota et al., 2018). The canga vegetation is composed of annual and perennial grasses and shrubs that contrast visibly with the surrounded tropical forest. Inside cangas, there are also small patches of forest (called *capão florestal*) where the soil is deeper. Cangas are found in the uplands of Serra Sul (the southernmost mountain) and Serra Norte (the northernmost mountain) in the National Forest of Carajás, as well in the bordering of the National Park of Campos Ferruginosos, which included two other uplands named Serra da Bocaina and Serra do Tarzan (median altitude of 607 m; Souza-Filho et al., 2019). It was already noted that at higher altitudes, the impact of climate change on biodiversity is likely more severe (Klanderud and Totland, 2005; Grabherr et al., 2010). Different species that occur along elevation gradients have changed their distributions in recent years by increasing their altitude in search of suitable climate zones (Kelly and Goulden, 2008; Lenoir et al., 2008; Steinbauer et al., 2018). However, such species may not always be able to timely adjust their distributions to climatically milder areas due to limitations in their dispersal capacity, which makes them particularly vulnerable (Colwell et al., 2008; Bertrand et al., 2011). Specifically to Amazon biome, high projected temperature change and drought intensification are expected to occur due to climate change (Intergovernmental Panel on Climate Change-IPCC [IPCC], 2014), with direct deleterious effects on biodiversity.

The main objective of this study is to evaluate if climate change will potentially impact plant species differently, considering their traits. We evaluated 608 plant species that occur in the naturally open areas of ferruginous outcrops in the National Forest of Carajás. We analyzed the following traits: pollination syndromes, habit and their main habitat. More specifically, the present study aims to determine: (i) the overall impact in the canga plant species; (ii) if different traits can be differently impacted by climate change; and (iii) the priority areas that could safeguard canga plant species and traits in the future against climate change.

## Materials and Methods

### Study Area

**FIGURE 1:**
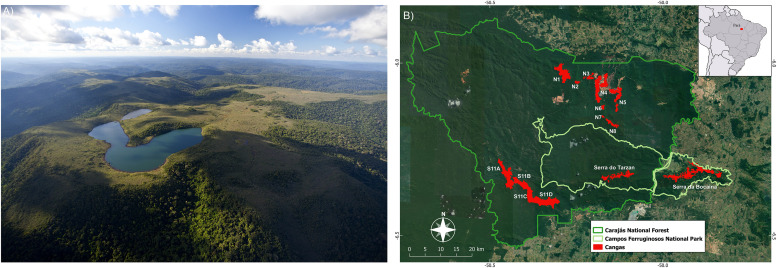
**(A)** Open vegetation occurring in upland outcrops (*canga*) (photo: João Marcos Rosa) **(B)** located in the National Forest of Carajás.

The canga sites analyzed here (Figure 1A) belong to the National Forest of Carajás (hereafter referred to as Carajás) and are located in the southeast of the state of Pará (Eastern Amazon Biome) (Figure 1B). Canga sites are located on discontinuous island-like habitats (Andrino et al., 2020), surrounded by Amazon forest. These areas of iron-rich substrate receive high solar irradiance, have more than 2,000 mm of rainfall annually, and undergo a pronounced dry season (Souza-Filho et al., 2016).

### List of Plant Species

Recently, the flora of cangas of Carajás was surveyed and reviewed by experts (Mota et al., 2018), which enables great accuracy for the analysis proposed here. The list of species surveyed is significant because it corresponds to almost 13% of the flora recorded for the state of Pará (Mota et al., 2018), the second largest state of Brazil and entirely within the Amazon biome. Some species of cangas are widely distributed, and few species with restricted distributions have been reported for Carajás (36 angiosperm species; Giulietti et al., 2019). The plant species of cangas totalize 855 angiosperm species in 110 families (Mota et al., 2018).

### Plant Species Habits and Pollination Syndromes

Habits of canga plant species were classified on five types (herbs, shrubs, trees, lianas, and parasites) and were defined in the study above mentioned (Mota et al., 2018). The pollination syndrome of those species was previously determined (Pinto et al., 2020) based on a group of plant characteristics (Supplementary Material A), following Faegri and Van Der Pijl (1979) and Rosas-Guerrero et al. (2014). Pollination syndrome for each plant species was determined based on available images, virtual herbaria sources, articles on the reproductive and flowering biology. It was possible to determine the syndrome of 771 species. Eight syndromes were analyzed: melittophily (pollinated by bees), phalaenophily (moths), psychophily (butterflies), cantharophily (beetles), entomophily (unspecified insects), ornithophily (birds), chiropterophily (bats), and anemophily (wind).

### Occurrences of Plant Species and Main Habitat

A database of the occurrences of each plant species of the cangas was organized by retrieving data from: (1) internal databases on species distribution; (2) online biodiversity databases, specifically the speciesLink and the Global Biodiversity Information Facility (GBIF)^1^; (3) consulting the specialized literature; and (4) through new fieldwork. We excluded repeated, dubious and incorrect occurrence points from online database. The total known geographical distribution of each species was used to run the models (see below) (the database of species occurrence can be found in Supplementary Material B).

As already said, some species are widely distributed, and although few species with restricted distributions have been reported for Carajás (36 species; Giulietti et al., 2019), they have not been addressed in detail here. As we aimed to determine if species occurring only (or preferentially) in the cangas will be significantly more affected than those occurring mainly in forest, the occurrences used for distribution modeling were overlaid on a land use map (ESA Climate Change Initiative—Land Cover led by Université Catholique de Louvain for 2017). The percentage of occurrences was calculated for the two main types of land cover analyzed here, namely, forest and open-area vegetation.

### Modeling of Species Distribution

The Biomod2 package (v.3.3-71; Thuiller, 2003) for R (R Development Core Team 2005) was used for species distribution modeling (SDM). Two algorithms were used: maximum entropy (MAXENT) (Phillips et al., 2006) and generalized linear model (GLM) (McCullagh and Nelder, 1989). Both were chosen due to their robustness and broad application (Li and Wang, 2013).

The environmental variables used in SDM were selected from the 20 less correlated bioclimatic layers (Aguirre-Gutiérrez et al., 2013) of the set available in WorldClim (Hijmans et al., 2005), which defines the mean temperature and precipitation data for 1970–2000 period, and altitude (5 arc min resolution). After selection, the following nine layers remained: Altitude, Mean Diurnal Range, Isothermality, Mean Temperature of Driest Quarter, Annual Precipitation, Precipitation of Driest Month, Precipitation Seasonality, Precipitation of Warmest Quarter, and Precipitation of Coldest Quarter. The predictive quality of the models was evaluated using the true skill statistic approach (TSS) (Allouche et al., 2006) with a minimum quality threshold ≥ 0.7, calculated by randomly partitioning the input data series into a quality testing set (25%) and a training set (75%), in successive rounds of cross-validation.

The climate change scenarios used for model projection refer to the estimates made for two future decades of short-medium term: 2050 and 2070. For both decades, we selected two IPCC scenarios of Representative Concentration Pathways (RCPs) (Intergovernmental Panel on Climate Change-IPCC [IPCC], 2014), which represent two different levels of radiative forcing (the difference between the insolation absorbed by the earth and the energy radiated back to space): 4.5 and 8.5 W/m^2^. The first scenario, 4.5, is more moderate (compared to extremes, RCP 2.6, and 8.5), whereas the second scenario, 8.5, represents more intensive climate change (Intergovernmental Panel on Climate Change-IPCC [IPCC], 2014). The scenarios were generated by two different institutes: Met Office Hadley Center (HadGEM2-ES, Hadley Global Environment Model 2—Earth System) and the National Center for Atmospheric Research (CCSM4, The Complete Coupled System).

The models obtained by the two institutes (HadGEM2-ES and CCSM4), the two algorithms (Maxent and GLM), and the different rounds were used to construct an ensemble forecast model to generate a single-scenario final model for each species. This model is even more robust because it groups the most important trends from the set of generated models based on the committee averaging method (Thuiller et al., 2009), producing normalized and comparable results among species.

In addition to the R package mentioned above, PostgreSQLs/PostGIS (The PostgreSQL Global Development Group) was used for the databases, together with the raster package (Hijmans and Etten, 2012) of R (R Development Core Team 2005), and the QGIS (Open Source Geospatial Foundation Project).

### Analysis of the Results

To answer our main questions, three analyses were conducted:

(i) To determine the overall impact in the canga plant species, we grouped the models of all plant species. The committee averaging method (Thuiller et al., 2009) was used. Species that potentially would not find suitable habitats in Carajás in the future under the analyzed climate change scenarios were identified. For this identification, species with no pixels with high-suitability (defined as ≥ 75% of suitability in a 0–100% range of occurrence probability) in any of the future scenarios analyzed in the study area were accounted.
(ii) To answer if different traits can be differently impacted by climate change, i.e., if the climate change impact will be potentially more accentuated in one or more pollination syndromes, habits or habitats of plant species, we used Generalized Linear Mixed Effects models with beta error distribution and logit link function using the glmmTMB package in R (Brooks et al., 2017). Our model uses the proportional loss of area with high suitability as the response variable, the different categories of syndrome, habit or habitat as predictors, and the different scenarios as random effects. The significance of the terms was determined by analysis of variance (package car, Fox and Weisberg, 2019). Finally, the estimated marginal means function was used to compare the proportional loss of area between the different categories (package emmeans; Lenth, 2019).
(iii) To determine the priority areas that could safeguard canga plant species and traits in the future against climate change, we forecasted the same models obtained in the item (i) for the surroundings of Carajás (following Giulietti et al., 2019; considering only Pará state).

## Results

Due to the scarcity of records of occurrence, not all species could be analyzed. Thus, models were obtained for 608 species (91 families; Supplementary Material C), belonging to eight syndromes, with a predominance of melittophily (bees; 53.5%) and, in the second place, anemophily (wind; 18.4%). Most of them are herbs (40%), followed by shrubs (27%), trees (18%), lianas (12%), and parasites (3%). A total of 483,632 occurrences were analyzed (Supplementary Material B). Almost half of the species (48%), considering the occurrence points obtained, is predominantly distributed in forest areas (>75% of the occurrence points) (Supplementary Material C). In regard specifically to Carajás, 257 plant species (42%) potentially will not find suitable habitats inside this protected area, as no pixel was detected there in any of the future scenarios analyzed (cells with zero value in Supplementary Material C).

### Overall Species and Most Affected Traits

**FIGURE 2:**
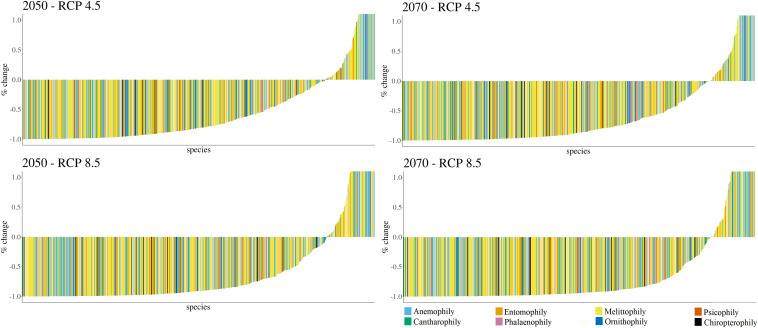
Area dynamics (in terms of future increase or decrease of suitable areas) for canga plant species due to climate change.

Potential loss of climatically suitable areas (i.e., areas with high climate resemblance considering current distribution and presenting high potential probability of species occurrence in the modeling processes) for most species was detected in all scenarios (Figure 2).

**FIGURE 3:**
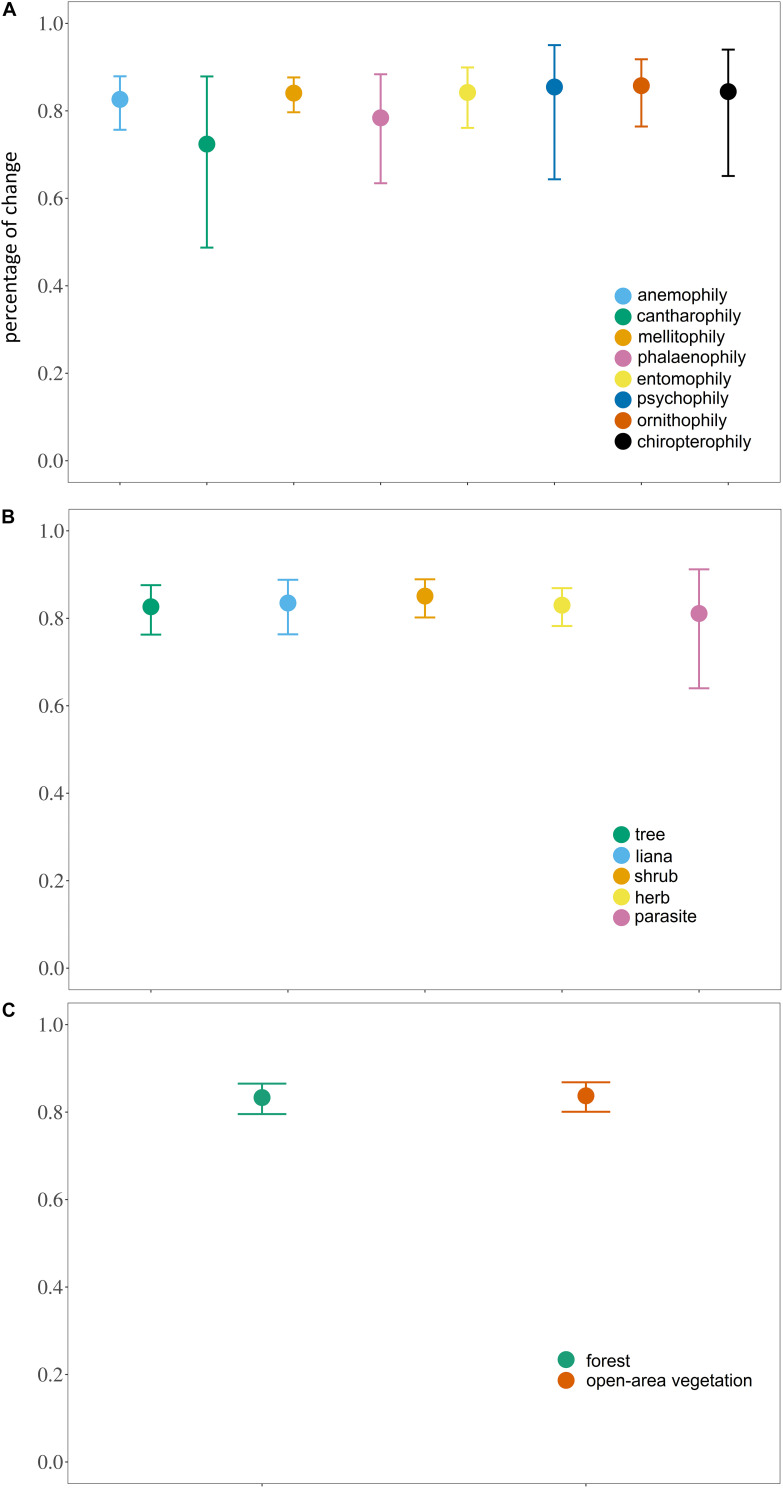
Estimated marginal mean (± 95% CI) of the proportional loss of areas with high habitat suitability (≥ 75%) for canga plant species with different **(A)** pollination syndromes; **(B)** habits and; **(C)** habitats.

However, no significant difference was observed within the different syndromes, habits or habitats (syndrome: *X*^2^ = 10.766, *df* = 7, *P* = 0.1492; habit: *X*^2^ = 6.2308, *df* = 5, *P* = 0.2844; habitat: *X*^2^ = 1.102, *df* = 1, *P* = 0.2938) (Figure 3).

### Priority Areas

**FIGURE 4:**
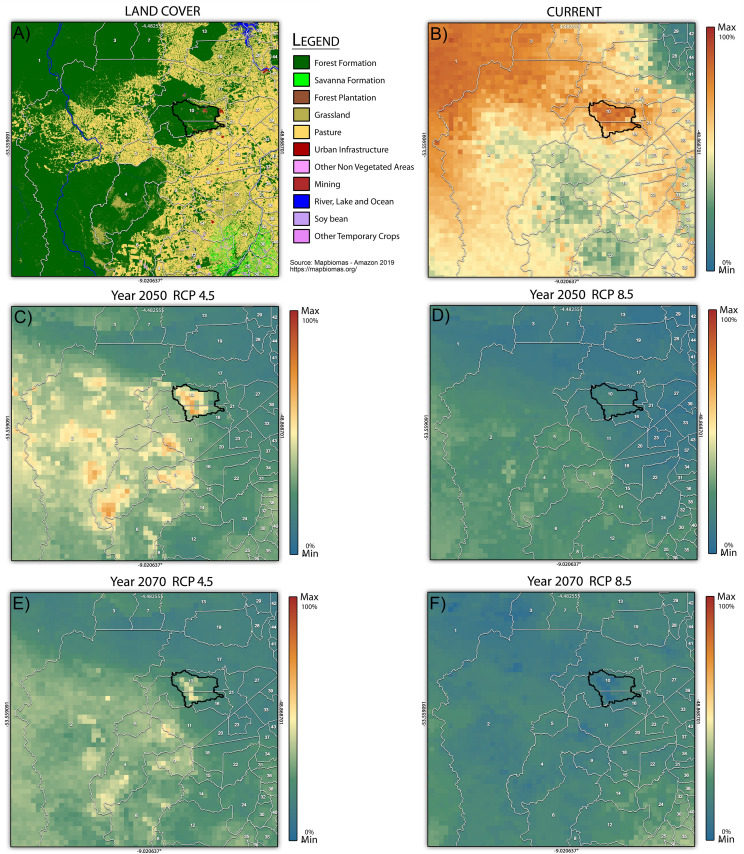
**(A)** The land cover of the projected area. **(B)** Current potential distribution and **(C–F)** future potential distribution considering the impact of climate change for all of the plant species of the cangas of the National Forest of Carajás (polygon in black). The scale variation corresponds to the percentage of species present per pixel (Max = 100%). White numbers refer to municipalities whose names can be found in Supplementary Material C.

Proximal areas western of Carajás were already deforested, but most of the areas to the southwest and northwest are still protected (Figure 4A). Considering the model’s projection (Figures 4B–F), we found that the areas to the southwest of study area (warmer colors in Figure 4) will be potentially suitable for the distribution of flora species in 2050 (Figure 4C) and 2070 (Figure 4E) under RCP4.5. Regarding year 2050 and RCP 8.5 (Figure 4D), most of the southwest region of the study area was also highlighted as a suitable area for the plant species analyzed. As for the scenario for 2070 and RCP8.5 (Figure 4F) few scattered areas will be suitable for the analyzed species, especially those from southwest and southeast of study area. Carajás presents some areas with high climatic suitability in the RCP4.5 scenario but, under RCP8.5, there is basically no area with high suitability.

## Discussion

According to the scenarios used, most of the 608 analyzed plant species potentially will face a severe reduction in their geographic distribution in the future decades due to climate change. From the total, 257 species (42%) may not occur in Carajás in future scenarios since there will be practically no area considered to be climatically suitable for them there. We found no significant difference in the impact of climate change on each pollination syndromes, habit or main habitat, i.e., the traits analyzed will be potentially equally affected. When all plant species are analyzed, final models showed that the areas with the highest potential suitability in the future for the analyzed species are located in the southwest of Carajás.

Future climate projections indicate a drastic reduction in plant species regardless the trait analyzed. As already noted, we were unable to find other studies that considered the same set of plant species traits used here under climate change. However, it was shown that other traits related to leaf and root (leaf mass per area, thicker leaves, root carbon content) were good predictors to species abundance shifts under temperature increase (Soudzilovskaia et al., 2013). Considering land cover change, morphological traits showed an important response on a long-term study (Kahmen et al., 2002). However, it is likely that multiple trait sets and other aspects of plant behavior have to be included when looking for unambiguous results (Díaz and Cabido, 1997). Analyzing complex responses, mediated by traits, to environmental change is not a trivial task, reinforcing the need for more robust theory (Kimball et al., 2016). However, this is an important effort since identifying such traits could allow generalized predictions for how species with similar traits will likely respond to climate change (Gornish and Prather, 2014).

The future projections of geographical distributions indicated that the areas to the southwest of the study area (with small variation, depending on the scenario) are probably more climatically stable, and could act as important areas for protecting the set of analyzed plant species. A previous study considering only the current potential distribution of canga plant species showed the western region as climatically suitable for these species in the current climate (Giulietti et al., 2019). In fact, the same region has been identified previously as important areas to protect other species such as bats (Costa et al., 2018; Ribeiro et al., 2018), birds (Miranda et al., 2019), and bees (Giannini et al., 2020) against climate change, since they potentially will provide suitable habitats for these animal species. It is noteworthy that some of these areas are relatively well-preserved, especially in the southernmost area. Previous studies have analyzed other open areas near Carajás and their importance for canga plant species conservation, such as Serra Arqueada (Devecchi et al., 2020) and Serra de Campos, both in the São Félix do Xingu municipality (Andrino et al., 2020), located in the southwestern region of our projected area. Other protected areas with high probability of future occurrence in the same southwestern region, which could be the target of future studies aiming conservation of canga’s plant species, are the Serra da Fortaleza (São Félix do Xingu), Serra do Bacajá (São Félix do Xingu), and Serra do Cubencranquém (Ourilândia do Norte). All of them were also emphasized in the previous study related to the potential current distribution of narrow-endemic canga plant species of Carajás (Giulietti et al., 2019).

Our results are restricted to habitats presenting a potential future high suitability for canga plant species, relying on the set of selected climatic variables, which participates in the fundamental niche of the species. However, according to ecological niche theories, the geographic distribution of a species is also influenced by other environmental, biological, and evolutionary factors (Soberón and Peterson, 2005). One important aspect is ecological interactions, and earlier studies considering the impact of climate change on interacting-animal species occurring in Carajás indicated the potential future loss of nectarivorous bat species (pollinators) in the order of 66% (Costa et al., 2018); nectarivorous birds in the order of 60% (Miranda et al., 2019); and bees in the order of 85% (Giannini et al., 2020). This last finding makes the results presented here even more noteworthy, since we found a high number of melittophilous plants in our study area. It is important to note that species-specific interaction data are still scarce, which hinders a detailed analysis. For example, plant-bee interactions in the *cangas* of Carajás have previously been evaluated (Pinto et al., 2020), but there is still no information about the plant species interactions with other animal groups. The global deficit of information on interactions has been previously emphasized (Hortal et al., 2015) and considering the Brazilian Amazon, few studies have evaluated interactions. Some studies analyzed plant species of economic interest pollinated by bees (Cavalcante et al., 2018; Krug et al., 2018; Beyerlein et al., 2019) or by complex systems involving multiple insects (Dattilo et al., 2012; Campbell et al., 2018). A small additional amount of data is available on interactions between plants and pollinators in the region, including bees (Moura et al., 2011; Novais and Absy, 2013; Ferreira and Absy, 2017; Oliveira et al., 2017; Milet-Pinheiro et al., 2018), beetles (Seymour and Matthews, 2006; Gottsberger and Webber, 2018), moths (Cruz-Neto et al., 2011), wasps (Nazareno et al., 2007), birds (Vicentini and Fischer, 1999), and bats (Gribel et al., 1999). None of the quoted studies analyzed the potential impact of climate change on those interactions. Other important feature considering the niche theory above mentioned (Soberón and Peterson, 2005) is related to the species’ ability to reach a site (i.e., mobility and dispersal). For example, enhancing the connectivity of natural habitats where these species occur now and in the future is an important feature that can mitigate the effects of climate change, as they improve the movement of species through suitable habitats (Miranda et al., 2021). Therefore, it is likely that our predictions may underestimate climate change impacts because interacting partners and species dispersion were not explicitly included in the models.

Some previous studies suggest a process of radical alteration of the Amazon biome, especially in the southern-southeastern portion, if no policy is implemented to control the additional loss of forest habitats due to deforestation (Nobre and Borma, 2009; Gomes et al., 2019; Brando et al., 2020). Deforestation intensifies global patterns of climate change effects, which may generate additional and localized variation in temperature and precipitation, creating an even greater impact on biodiversity (Barkhordarian et al., 2019) and further forest dieback (Staal et al., 2020). In fact, a local temperature increase of 1°C and a reduction of 0.8 mm rainfall/day was cited as a consequence of deforestation in the Amazon (Pitman and Lorenz, 2016). Carajás is surrounded by a region that presents high rates of deforestation (Souza-Filho et al., 2016). Our models showed that the proximal areas to the west of Carajás will likely be important areas considering climate change, but most of them are already deforested. We hope that our study can foster restoration strategies on these areas. Our results can also help on providing a useful list of species that are potentially more resistant to climate change, and that can be prioritized aiming to potentially increase the medium- and long-term success of restoration strategies (Harris et al., 2006). This list can also help in the selection of species that can provide resources for different animal groups (Martins and Antonini, 2016; Giannini et al., 2017), which could assist in the restoration of interactions and ecosystem functions in the eastern Amazon. Such initiatives can help in the conservation and protection of biodiversity against the impacts of climate change.

## Data Availability Statement

The original contributions presented in the study are included in the article/Supplementary Material, further inquiries can be directed to the corresponding author/s.

## Author Contributions

TG and WC: conceptualization. WC, CP, MW, DZ, and AG: data curation. WC, AA, and LM: formal analysis. TG: funding acquisition. TG, AA, and LM: writing—original draft. All authors: review and editing.

## Conflict of Interest

The authors declare that the research was conducted in the absence of any commercial or financial relationships that could be construed as a potential conflict of interest.

## Publisher’s Note

All claims expressed in this article are solely those of the authors and do not necessarily represent those of their affiliated organizations, or those of the publisher, the editors and the reviewers. Any product that may be evaluated in this article, or claim that may be made by its manufacturer, is not guaranteed or endorsed by the publisher.

## Footnotes

1
https://doi.org/10.15468/dl.vytofp
